# In Situ Polycondensation Synthesis of NiS-g-C_3_N_4_ Nanocomposites for Catalytic Hydrogen Generation from NaBH_4_

**DOI:** 10.3390/nano13050938

**Published:** 2023-03-05

**Authors:** Alhulw H. Alshammari, Khulaif Alshammari, Turki Alotaibi, Majed Alshammari, Sultan Alhassan, Taha Abdel Mohaymen Taha

**Affiliations:** Physics Department, College of Science, Jouf University, Sakaka P.O. Box 2014, Saudi Arabia

**Keywords:** carbon nitride, NiS, hydrogen energy, methanolysis, NaBH_4_

## Abstract

The nanocomposites of S@g-C_3_N_4_ and NiS-g-C_3_N_4_ were synthesized for catalytic hydrogen production from the methanolysis of sodium borohydride (NaBH_4_). Several experimental methods were applied to characterize these nanocomposites such as X-ray diffraction (XRD), Fourier transform infrared spectroscopy (FTIR), and environmental scanning electron microscopy (ESEM). The calculation of NiS crystallites revealed an average size of 8.0 nm. The ESEM and TEM images of S@g-C_3_N_4_ showed a 2D sheet structure and NiS-g-C_3_N_4_ nanocomposites showed the sheet materials that were broken up during the growth process, revealing more edge sites. The surface areas were 40, 50, 62, and 90 m^2^/g for S@g-C_3_N_4_, 0.5 wt.% NiS, 1.0 wt.% NiS, and 1.5 wt.% NiS, respectively. The pore volume of S@g-C_3_N_4_ was 0.18 cm^3^, which was reduced to 0.11 cm^3^ in 1.5 wt.% NiS owing to the incorporation of NiS particles into the nanosheet. We found that the in situ polycondensation preparation of S@g-C_3_N_4_ and NiS-g-C_3_N_4_ nanocomposites increased the porosity of the composites. The average values of the optical energy gap for S@g-C_3_N_4_ were 2.60 eV and decreased to 2.50, 2.40, and 2.30 eV as the NiS concentration increased from 0.5 to 1.5 wt.%. All NiS-g-C_3_N_4_ nanocomposite catalysts had an emission band that was visible in the 410–540 nm range and the intensity of this peak decreased as the NiS concentration increased from 0.5 to 1.5 wt.%. The hydrogen generation rates increased with increasing content of NiS nanosheet. Moreover, the sample 1.5 wt.% NiS showed the highest production rate of 8654 mL/g·min due to the homogeneous surface organization.

## 1. Introduction

Research on graphitic carbon nitride (g-C_3_N_4_) has received a lot of attention due to its structure and substantial chemical and physical characteristics, such as superior electrical conductivity and high mechanical strength [1,2,3]. Moreover, the use of g-C_3_N_4_ in photocatalysis, electrocatalysis, photovoltaic devices, and bioimaging applications has a lot of potential [4,5,6]. It is composed of several abundant elements and is the most stable allotrope of carbon nitrides under ambient conditions. Graphite and g-C_3_N_4_ have a similar structure, however one by one, nitrogen and carbon atoms make up the hexatomic ring. A significant planer network structure is developed by the covalent connections that are formed by each carbon atom with three nitrogen atoms. The sp^2^ hybridized C and N atoms are organized in a six-member stalked ring; providing the semiconductor properties of g-C_3_N_4_. In the optical range of 260–320 nm, g-C_3_N_4_ has a significant UV–Vis absorption peak. The π-π* electron transfer for g-C_3_N_4_ with s-triazine rings is responsible for the absorption peak, which is located at about 250 nm. Moreover, the n-π* electron transfer involving a lone pair of electrons on nitrogen atoms in the g-C_3_N_4_ produced the absorption peak at 320 nm [7,8]. The electrical features and surface chemical properties of g-C_3_N_4_ doped with heteroatoms (such as oxygen, sulfur, phosphorous, and boron) can be tuned, which is advantageous for expanding their applications in bioimaging and biosensing. For catalytic usage, g-C_3_N_4_ has enhanced thermal stability because of higher durability and non-volatility up to 600 °C even in the air, according to the thermogravimetric analysis [8,9,10]. However, the limited solubility of g-C_3_N_4_ in organic solvents resists the processing of this material.

There is a potential use for metal sulfides in a variety of industries, including supercapacitors, catalysis, energy conversion, and biomedicine [11,12]. Due to their many structural forms, several metal sulfides have been investigated and widely used. Metal sulfide nanoparticle applications are influenced by characteristics including size, shape, surface area, and morphology. A well-known metal sulfide called nickel sulfide has been suggested as a promising material because of its nontoxicity, abundant mineral resources, and high stability for various energy storage and energy conversion applications [13,14]. The metallic structure of NiS provides high electrical conductivity, and this high conductivity enhances interfacial charge transfer and carrier separation. In order to facilitate interfacial charge transfer and separation, the NiS catalyst can serve as an electron-trapping agent and a site for the generation of H_2_. Nanoscale nickel sulfide with a specified surface charge and functionality may be of interest for catalytic applications [15]. As a result, it is crucial to develop a precise preparation approach to couple nickel sulfides with the g-C_3_N_4_ nanosheet. Accordingly, the nanocomposite of NiS/g-C_3_N_4_ is expected to have a large surface area and more active sites for hydrogen catalytic performance.

Over the past few years, numerous efforts have focused on the synthesis of g-C_3_N_4_. Heating the carbon- and nitrogen-containing precursors was the primary method of producing graphite carbon nitride [16]. Cyanamide, cyanuric chloride, ethylenediamine with carbon tetrachloride, and melamine are among the most frequently utilized substances in synthesis [17]. Additionally, the usage of templates allows for the synthesis of materials with regular structures [18]. The ability to electrostatically self-assemble composite structures with negatively surface-charged materials to function because cocatalysts are one of the main benefits of the positively charged g-C_3_N_4_ material [19]. The surface of g-C_3_N_4_-TiO_2_ was modified with phosphorous for hydrogen generation from NaBH_4_ methanolysis [20]. The data showed a hydrogen evolution rate of 14,750 mL/g·min. Furthermore, the activation energy for hydrolysis was estimated to be 36.17 kJ mol^−1^. Kottaikalai Ganesan et al. [21] reported utilizing phosphorylated silica (SP-PA) particles for the catalytic hydrolysis of sodium borohydride to produce hydrogen. In comparison to silica particles (133 mL min^−1^g^−1^ of catalyst), SP-PA particles generate hydrogen at a rate of 762.4 mL min^−1^g^−1^. The remarkable catalytic activity of SP-PA particles is indicated by the computed activation energy of 29.92 kJ mol^−1^ for NaBH_4_ hydrolysis. Fanghui Wang et al. [22] synthesized Co-P/CNTs-Ni foam for catalytic hydrogen generation at methanolysis of NaBH_4_. The dandelion-like structure of the Co-P/CNTs-Ni foam catalysts was maintained and produced a maximum amount of hydrogen (2430 mL min^−1^g^−1^) at a temperature of 298 K. The data showed that NaBH_4_ hydrolysis has an activation energy of 49.94 kJ mol^−1^. Another study used calcination and hydrothermal techniques to generate a hybrid g-C_3_N_4_-SiO_2_-N composite [23]. The experiments conducted using an NaBH_4_ content of 0.25 g revealed a maximal hydrogen evolution rate of 11,400 mL min^−1^g^−1^. According to calculations, the hydrolysis of NaBH_4_ has an activation energy of 33.2 kJ mol^−1^. Cafer Saka [24] prepared catalysts of sulfur and nitrogen-doped metal-free microalgal carbon that are very active for the dehydrogenation of sodium borohydride in methanol. Their study achieved a maximal hydrogen generation rate of 26,000 mL min^−1^g^−1^. Meanwhile, the activation energy for hydrolysis was 10.59 kJ mol^−1^. Highly dispersed CoB alloys implanted on MOF-74-derived graphene nanosheets were synthesized using the chemical reduction technique [25]. The catalytic hydrogen evolution measurements showed a value of 7937 mL min^−1^g^−1^ for the hydrolysis of NaBH_4_. Furthermore, the investigation of activation energy for hydrolysis was evaluated as 38.8 kJ mol^−1^.

Graphitic carbon nitride suffers from low conductivity and a small electroactive surface area. Low carrier mobility and excessive bulk recombination are the main reasons limiting the efficiency of g-C_3_N_4_. Therefore, researchers proposed many techniques to improve conductivity and surface area by elemental doping, converting into nanosheets, and combining with metal nanoparticles and other carbon nanomaterials. This in turn will suppress the electron–hole recombination. The in situ polycondensation process can produce metal/g-C_3_N_4_ nanocomposites. Meanwhile, this synthesis procedure enables the direct formation of nanocomposites as well as microstructure control. Moreover, this technique has excellent aggregate elimination/reduction along with ideal and reproducible properties.

The current study aims to find an appropriate precursor, morphology, exfoliation condition, and fabrication processes for g-C_3_N_4_ in order to improve the catalytic activity. Furthermore, NiS nanostructures can be integrated in situ into the g-C_3_N_4_ nanosheet to increase the electroactive surface area and electrical conductivity. The in situ polycondensation process using different ratios of nickel chloride and thiourea at 550 °C for 120 min synthesized the NiS-g-C_3_N_4_ nanocomposite samples. The structural measurements for these nanocomposites was conducted using XRD, FTIR, and ESEM techniques. Moreover, an extensive study of the methanol hydrolysis of sodium borohydride was completed. Finally, the hydrogen catalytic efficiency of prepared materials was examined at methanolysis of NaBH_4_. The hydrogen generation rates increased with increasing NiS nanosheet content. Moreover, the sample 1.5 wt.% NiS showed the highest production rate of 8654 mL/g·min.

## 2. Experimental

Loba Chemi, Mumbai, India supplied the chemicals (nickel chloride hexahydrate, thiourea, and sodium borohydride). The absolute methanol was provided by Sigma-Aldrich, Darmstadt, Germany. All provided chemicals were directly used without extra purification.

The bulk S@g-C_3_N_4_ was synthesized by heating 10.0 g of thiourea powder in a porcelain crucible covered with a lid in the muffle furnace at 550 °C (ramping rate = 3.0 °/min) and maintaining the temperature for 2 h. The yellow solid mass in a crucible was then allowed to cool to room temperature. Using an agate mortar, the resultant bulk S@g-C_3_N_4_ was crushed into a fine powder. The in situ polycondensation process using different ratios of nickel chloride and thiourea at 550 °C for 120 min synthesized NiS-g-C_3_N_4_ nanocomposite samples. In a typical synthesis, 10 g of thiourea powder and 0.5, 1.0 and 1.5 wt.% of nickel chloride were ground in an agate mortar for 30 min. After that, the powder was transferred to porcelain crucibles covered with a lid inserted inside a muffle furnace. The furnace operated at 550 °C for 120 min at a heating rate of 3.0 °C/min. The obtained nanocomposite samples were allowed to cool and then ground.

X-ray diffraction studies can analyze structural factors such as crystallinity, grain size, strain, phase composition, and structural defects. The XRD spectra were recorded from a Shimadzu XRD 700 instrument utilizing a Cu_kα_ wavelength of 1.54056 Å. Cu_kα_ radiation was created by using a Cu source as an X-ray source. The data scans were collected at the 2Theta range 5.0–80° with a count rate of 0.2°/min. The crystal structure was identified by comparing the diffraction pattern of the synthesized nanocomposite with the JCPDS files in the database. FTIR data were collected using a Shimadzu FTIR spectrometer—100 Tracer. The frequency ranges that can be examined are generally in the 4,000,399 cm^−1^ range. The sensitive characterization instrument ESEM exposes surface morphology and when coupled with an EDX (energy dispersive X-ray analysis) accessory, determines the elemental composition of materials. High-resolution 3D micrographs of the morphology were provided utilizing the ESEM technique. ESEM images and EDX data were acquired using a Thermo Fisher Quattro environmental scanning electron microscope (ESEM). Transmission electron microscopy (TEM) has become an essential tool in medical, biological, and materials’ research because of its high magnifications. An investigation of TEM microscopy was carried out using a Thermo Fisher Scientific Talos F200i TEM/STEM electron microscope. The most generally used technique for calculating the specific surface area of produced material is the Brunauer–Emmett–Teller (BET) method. The BET technique involves the multi-layer adsorption of chemically inert N_2_ gas with relative pressure and gas volume adsorbed in cm^3^/gm. The samples were degassed at 100 °C overnight to remove trapped moisture molecules. The samples were subjected to N_2_ gas at 77 K to record the adsorption–desorption isotherm on NOVA 4200e surface area analyzer. As a significant characterization tool in the field of photocatalysis, UV–Vis spectroscopy is a non-destructive method for analyzing optical characteristics such as absorbance, reflectance, transmittance, and bandgap energy that are related to the chemical composition of the material. In order to move electrons from their ground state to their excited state for electronic spectroscopy, it is necessary to absorb photons in the UV–visible region of the spectrum. The UV–Vis spectra were recorded on Agilent Cary 60 Spectrophotometer. The photoluminescence (PL) approach has been widely applied in the field of photocatalysis to study surface processes. Photoluminescence (PL) spectra at room temperature were obtained using a Cary Eclipse fluorescence spectrometer with a 350 nm excitation wavelength.

The hydrogen catalytic efficiency of prepared materials was evaluated as follows. Typically, 20 mg of the nanocomposite sample was mixed with 0.25 g of NaBH_4_ and 10 mL of methanol was added without stirring. The hydrogen gas volume was recorded via the water displacement method. The measurements were completed at a temperature of 30 °C.

## 3. Results and Discussion

**Figure 1:**
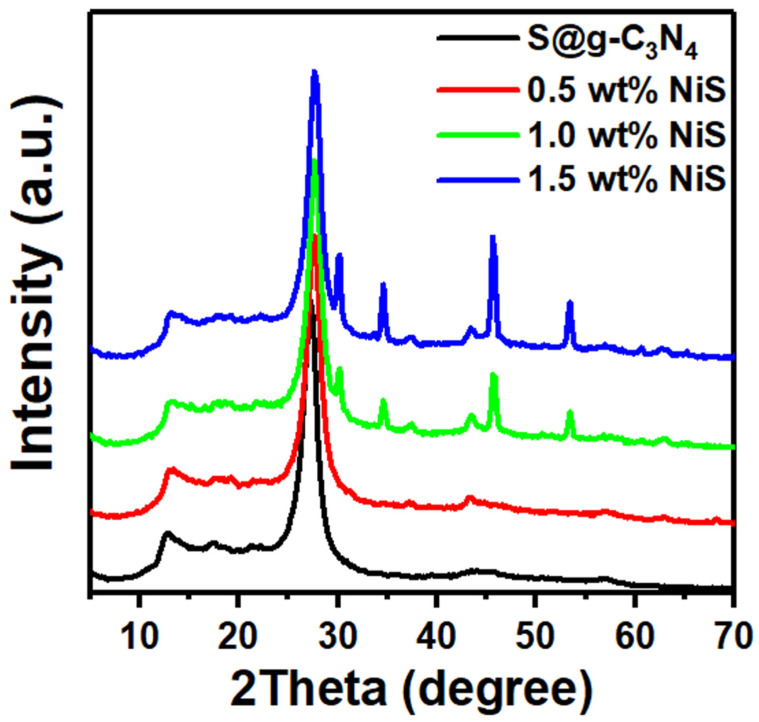
XRD data of S@g-C_3_N_4_ and NiS-g-C_3_N_4_ nanocomposites.

XRD is a non-destructive analytical method that provides details of the physical characteristics and crystalline structure of materials. The results of XRD data for the synthesized S@g-C_3_N_4_ and NiS-g-C_3_N_4_ nanocomposite catalysts are presented in Figure 1. The catalyst included a variety of compounds which were discovered using XRD analysis. The presence of two major peaks at 13.08° and 27.20° were observed in the spectrum of S@g-C_3_N_4_. The small diffraction peak at 13.08° matched a (100) plane with a distance of 0.676 nm [26,27]. The highest diffraction peak with reflection (002) was a typical interlayer stacking peak for graphitic C_3_N_4_ material and located at 2θ = 27.20°, which occurs because the atomic radius of sulfur is larger than carbon and nitrogen. Further, small diffraction peaks of sulfur were observed in the XRD spectrum of S@g-C_3_N_4_ [28]. For NiS-g-C_3_N_4_ at 0.5, 1.0, and 1.5 wt.%, the peak (002) moved to 27.55°, 27.66° and 27.64°, respectively. The positions of these reflections shifted to higher angles and thus lowered d-values with increase in NiS content. This indicates a structural contraction along the layer-stacking direction, presumably because of a more extended condensation of the carbon nitride chains [29]. Moreover, this shift occurs because of the reduced size (layer thickness) in carbon nitride sheets [30].

The reflections of NiS are located at 30.24°, 34.54°, 45.65°, 53.34°, 60.71°, and 62.94° that corresponds to (100), (101), (102), (110), (103), and (200) of hexagonal crystal structure (ICDD No. 50-1791). The synthesis of the NiS-g-C_3_N_4_ nanocomposites is confirmed by the existence of the two phases. Meanwhile, the crystallite size (*D*) is inversely proportional to the diffraction peak broadening (*β*) as proposed by the Scherer equation [31,32]:

(1) $$D=\frac{0.9\lambda }{\beta \cos\theta }$$

Accordingly, the calculations of b at the diffraction peak with reflection (002) for S@g-C_3_N_4_ decreased as the NiS concentration increased from 0.5 to 1.5 wt.%. This reveals the small crystallite domains of S@g-C_3_N_4_. The calculation of NiS crystallites reveals an average size of 8.0 nm for 0.5 wt.% NiS, 1.0 wt.% NiS, and 1.5 wt.% NiS. Moreover, the shift of peak position for the (002) plane after the growth of NiS at different content (0.0–1.5 wt.%) confirms the successful formation of nanocomposites.

**Figure 2:**
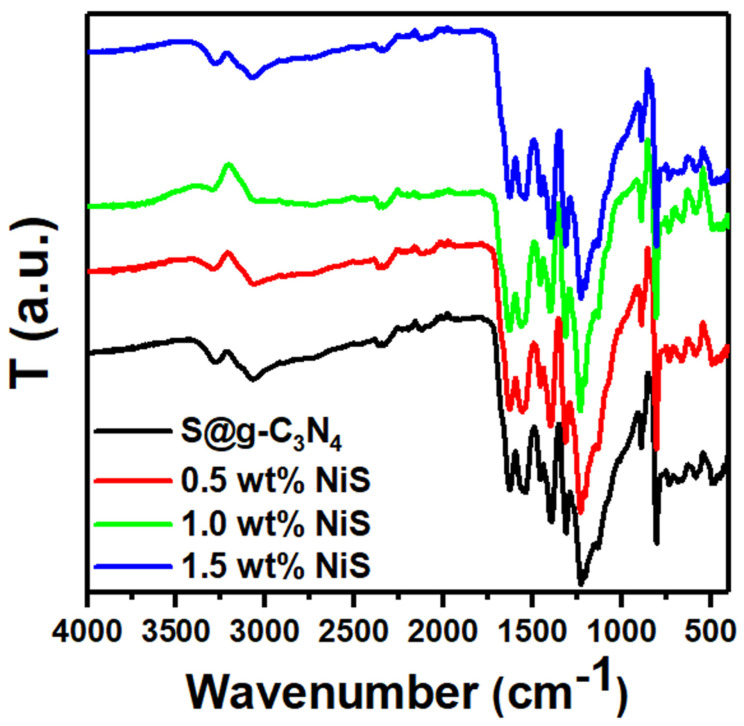
FTIR data of S@g-C_3_N_4_ and NiS-g-C_3_N_4_ nanocomposites.

Figure 2 shows the FTIR spectra of S@g-C_3_N_4_ and NiS-g-C_3_N_4_ nanocomposite samples. The vibrations of C-O stretching and C-OH stretching for the S@g-C_3_N_4_ sample located at 1010 and 1132 cm^−1^ [33]. This result indicates the existence of both hydroxyl (C-OH), carbonyl (C=O), and carboxylic (COOH). The vibrations of N-H stretching or the H_2_O adsorption showed two peaks at 3100–3300 cm^−1^ [34]. The absorption bands between 1229 and 1628 cm^−1^ observed in the spectrum of S@g-C_3_N_4_ correspond to the typical stretching modes of CN heterocycles [35]. It was determined that the physically adsorptive CO_2_ from the environment was responsible for a weak band at 2336 cm^−1^ [30]. Moreover, another weak peak connected to the C=N bond was seen at 2170 cm^−1^ [36].

The medium intense sharp band at around 804 cm^−1^ suggests the samples consist of triazine or heptazine building blocks [37]. The bands at 1205 and 1311 cm^−1^ indicate the presence of C–N (sp^3^) and C-N(-C)-C bonds [38]. Moreover, the positions of these peaks slightly shift to a higher wavenumber after the growth of NiS at 1.5 wt.%. These outcomes provide evidence of the successful preparation of NiS-g-C_3_N_4_ nanocomposites and agree with the XRD investigations.

**Figure 3:**
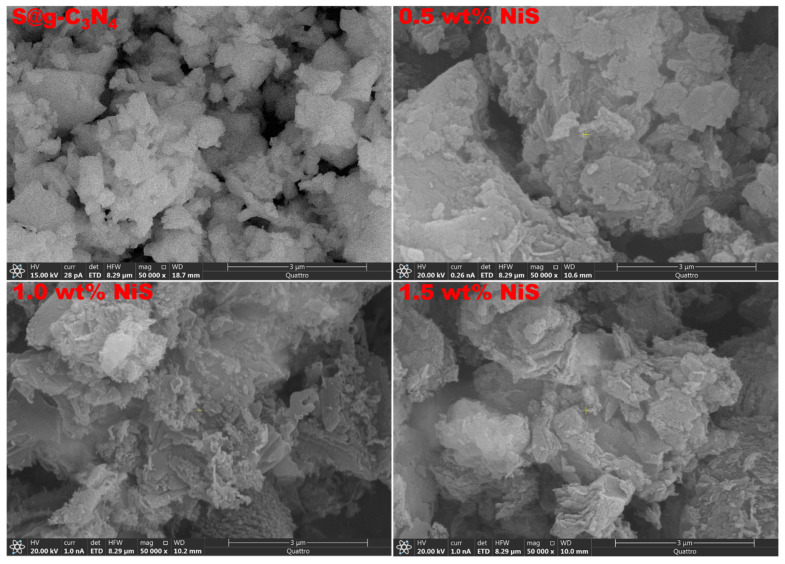
ESEM micrographs of S@g-C_3_N_4_ and NiS-g-C_3_N_4_ nanocomposites.

The morphology and structure of the synthesized S@g-C_3_N_4_ and NiS-g-C_3_N_4_ nanocomposite samples were investigated by ESEM images. In Figure 3, S@g-C_3_N_4_ shows that a 2D sheet structure was achieved for g-C_3_N_4_ materials. NiS-g-C_3_N_4_ nanocomposites showed the sheet materials were broken up during the growth process in Figure 3, revealing more edge sites. This aligns with the XRD result showing that NiS-g-C_3_N_4_ is less ordered and crystalline by virtue of the broader spectral peaks.

The EDX spectra displayed in Figure S1 (Supplementary Materials) of the NiS-g-C_3_N_4_ nanocomposite samples confirm the presence of all the elements supposed. Table 1 also displays the weight percent of the elements found on the samples’ surfaces. Moreover, Figure S1 shows no identifiable peaks for any other elements besides Ni, S, C, and N, demonstrating that the NiS-g-C_3_N_4_ nanocomposite samples are of high purity.

**Figure 4:**
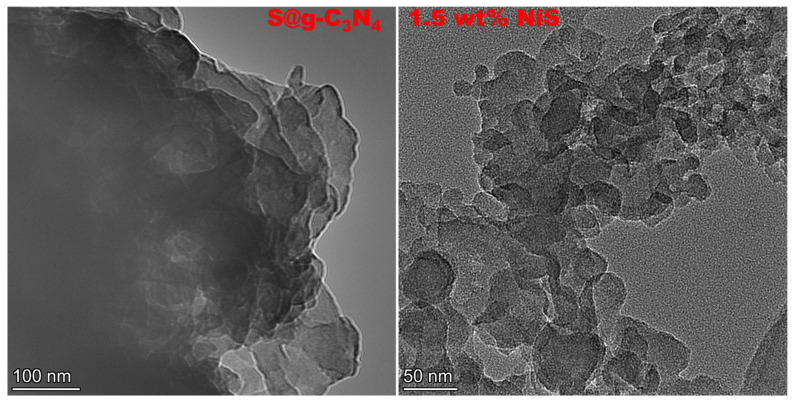
TEM micrographs of S@g-C_3_N_4_ and 1.5 wt.% NiS nanocomposites.

TEM images of S@g-C_3_N_4_ and 1.5 wt.% NiS nanocomposite samples are shown in Figure 4. The lamellar structure with sheet morphology is seen in the images. The image of S@g-C_3_N_4_ reveals stack layers that are connected with XRD and ESEM measurements. Moreover, the sheet materials in 1.5 wt.% NiS nanocomposite sample were broken up throughout the growing process.

**Figure 5:**
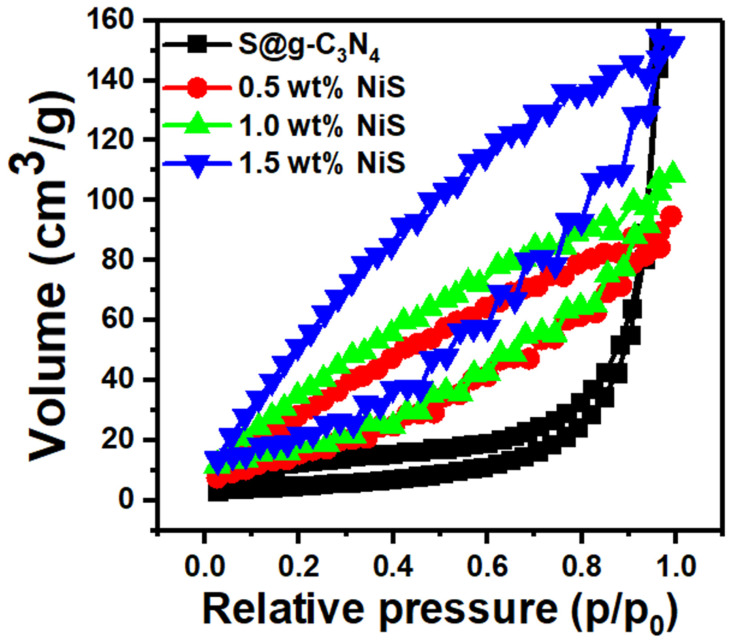
Nitrogen adsorption–desorption isotherm of S@g-C_3_N_4_ and NiS-g-C_3_N_4_ nanocomposites.

An adsorbent surface area, which is directly related to the number of active sites for adsorption, has a significant impact on its catalytic activity. We measured surface area using the N_2_ adsorption–desorption isotherm at 77 K for the S@g-C_3_N_4_ and NiS-g-C_3_N_4_ nanocomposite samples (Figure 5). All the samples exhibited type IV isotherm with no saturation implying mesoporous nature. BET plots give specific surface areas of 40, 65, 66, and 83 m^2^/g for S@g-C_3_N_4_, 0.5 wt.% NiS, 1.0 wt.% NiS, and 1.5 wt.% NiS, respectively. The surface area represents the effect of the in situ polycondensation preparation of S@g-C_3_N_4_ and NiS-g-C_3_N_4_ nanocomposites. The BJH pore volume of S@g-C_3_N_4_ is 0.18 cm^3^, which increases to 0.20 cm^3^ in 1.5 wt.% NiS owing to the incorporation of NiS into the nanosheet. We found that the in situ polycondensation preparation of S@g-C_3_N_4_ and NiS-g-C_3_N_4_ nanocomposites increased the porosity of the composites, which allowed for more interaction with ions and faster electron transport for catalytic activity [39].

**Figure 6:**
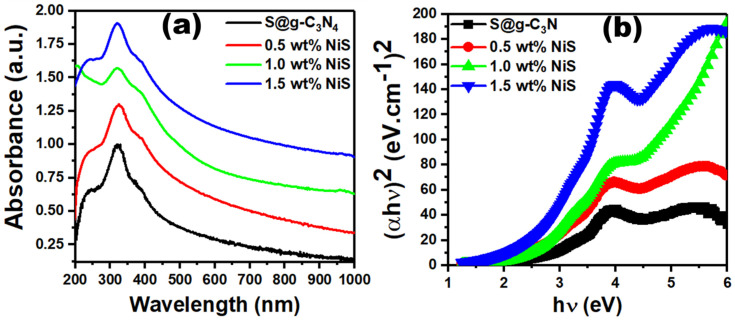
Graphs of (**a**) absorbance vs. wavelength and (**b**) (ahn)^2^ vs. photon energy for S@g-C_3_N_4_ and NiS-g-C_3_N_4_ nanocomposites.

The absorbance properties of S@g-C_3_N_4_ and NiS-g-C_3_N_4_ nanocomposite samples were measured with the help of UV–visible spectroscopy as shown in Figure 6a. The absorbance curve displays a significant absorption band centered around 322 nm that corresponds to n→π* electronic transitions. Heterocyclic aromatics showed the band gap absorption around 400 nm that corresponds to the characteristic π-π* transitions [8]. It is also expected that the disorder in nanocomposites will result in separated electron and hole states with energies in the band gap, causing the Urbach tail in the optical absorption spectrum, which broadens the absorption even more. Moreover, a shoulder appeared at 400 nm that showed a red shift when NiS content varies from 0.0–1.5 wt.%. The red shift in adsorption revealed the ease of production of photo-induced electrons and holes.

The optical energy gap (*E_opt_*) is an important parameter to estimate the electronic structure of the S@g-C_3_N_4_ and NiS-g-C_3_N_4_ nanocomposites. *E_opt_* is calculated by evaluation of the straight lines intercept at zero photon absorption from the plots of (*αhν*)^2^ vs. photon energy (*hν*) shown in Figure 6b as follows [40,41,42]:

(2) $$\alpha h\upsilon =k{\left(hv-E_{g}\right)}^{0.5}$$

The average values of the optical energy gap for S@g-C_3_N_4_ were 2.60 eV that decreased to 2.50, 2.40, and 2.30 eV as the NiS concentration increased from 0.5 to 1.5 wt.%. This decrease in the energy gap is explained by the development of additional energy levels or changes in the g-C_3_N_4_ electronic structure [43,44].

**Figure 7:**
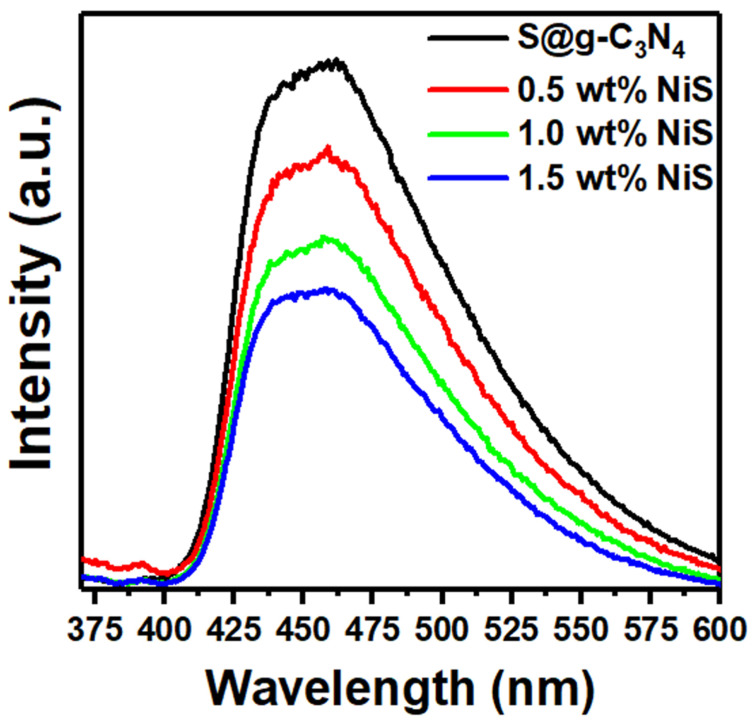
PL spectra for S@g-C_3_N_4_ and NiS-g-C_3_N_4_ nanocomposites.

Physical and chemical characteristics of materials are measured in photoluminescence by employing photons to produce excited electronic states in the material system and evaluating the optical emission when these states relax. This in turn induces electron-hole pairs that recombine after a lifetime in excited states. The key factors influencing a catalyst’s capacity to catalyze a reaction are the electrical and structural defects as well as the recombination of electron-hole pairs [45]. Figure 7 shows the emission spectra of S@g-C_3_N_4_ and NiS-g-C_3_N_4_ nanocomposites. All NiS-g-C_3_N_4_ nanocomposite catalysts have an emission band that is visible in the 410–540 nm range and is composed of n-π* transitions [46]. The intensity of this peak decreased as the NiS concentration increased from 0.5 to 1.5 wt.%. This could be because electron–hole pairs are produced quickly while the pair recombination process is delayed. Further, the photo-induced electron–hole pair can transfer easily at the interface of NiS/g-C_3_N_4_ nanocomposite. As a result, we expect that the nanocomposite sample 1.5 wt.% NiS will show high catalytic performance concerning the other samples.

In self-hydrolysis, sodium borohydride solutions become chemically stable and do not produce substantial quantities of H_2_ at ambient conditions. In pure water, sodium borohydride undergoes self-hydrolysis, consuming H_3_O^+^ ions, and causing a pH rise that lowers the rate at which hydrogen is produced [47]. Methanol is one of the highest reactivity reagents toward sodium borohydride and is the lightest alcohol, which makes it a suitable alternative for water in the reaction that produces hydrogen. Another benefit of employing methanol is that it lowers the reactant mixture’s freezing point, making it feasible to generate hydrogen at temperatures lower than 273 K with rapid hydrogen synthesis and quick reaction initiation—impossible when using pure water. Moreover, methanol regeneration may be employed as a possible high gravimetric density hydrogen storage device, and sodium borohydride methanolysis has been presented as a practical process for hydrogen production at low temperatures [48].

According to the Langmuir–Hinshelwood mechanism, catalysts whose surfaces are linked with the amino group effectively contribute to the hydrolysis or methanolysis of NaBH_4_ and for hydrogen generation. Meanwhile, methanol and NaBH_4_ molecules adsorb on the catalyst’s surface [49,50]. On the other hand, Michaelis–Menten stated that the active sites of the catalyst adsorb NaBH_4_ without methanol [51,52]. The aforementioned information leads to the conclusion that catalyst surface characteristics are crucial for the evolution of hydrogen gas.

**Figure 8:**
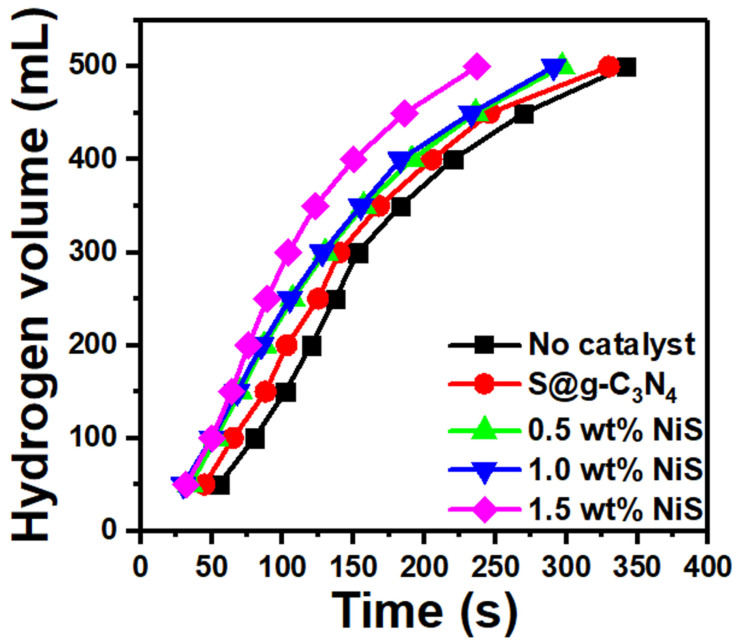
Hydrogen production from methanolysis of NaBH_4_ of S@g-C_3_N_4_ and NiS-g-C_3_N_4_ nanocomposites.

Nanocomposite catalysts are employed to accelerate the kinetics of sodium borohydride hydrolysis in stable solutions, resulting in a significant increase in hydrogen production. The particle size and degree of dispersion are other factors that affect a catalyst’s activity. In order to speed up the reaction and lower the catalyst loading, small particle size and excellent dispersion encourage extensive catalyst interaction with the NaBH_4_ solution. A methanolysis experiment was performed in order to check the role of S@g-C_3_N_4_ and NiS-g-C_3_N_4_ nanocomposites in the hydrogen evolution from NaBH_4_. The data were recorded at 30 °C and plotted in Figure 8. S@g-C_3_N_4_ and NiS-g-C_3_N_4_ nanocomposites were added and led to an increase in the maximum quantity of hydrogen. The sample 1.5 wt.% NiS showed the fastest hydrogen generation performance. In methanol, the NaBH_4_ material broke down into Na^+^ and ${\mathrm{BH}}_{4}^{-}$ ions. Moreover, the surface of S@g-C_3_N_4_ and NiS-g-C_3_N_4_ nanocomposites adsorbed ${\mathrm{BH}}_{4}^{-}$ ions. The efficient catalyst adsorbs more ${\mathrm{BH}}_{4}^{-}$ ions in a short time and thus produces more hydrogen.

The rate of hydrogen production greatly determines the efficiency of the catalyst to speed up the reaction. Hydrogen evolution rates (*r*) of the S@g-C_3_N_4_ and NiS-g-C_3_N_4_ nanocomposites are calculated with help of the following equations using the H_2_ volume (*V*), the mass of catalyst (*m_cat_*), and time of reaction (*t*) [20,53]:

(3) $$r=\frac{V}{t\cdot m_{cat}}$$

**Figure 9:**
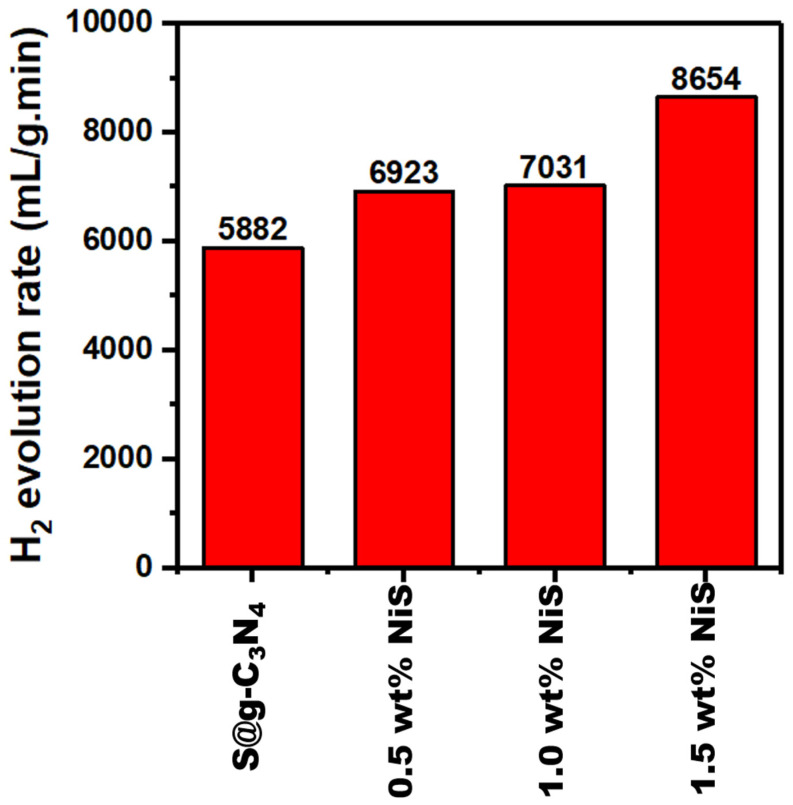
H_2_ production rates for S@g-C_3_N_4_ and NiS-g-C_3_N_4_ nanocomposites.

The hydrogen evolution curves displayed in Figure 8 were used to calculate the hydrogen generation rate. The calculated production rates are plotted in Figure 9 for the S@g-C_3_N_4_ and NiS-g-C_3_N_4_ samples. The hydrogen generation rates increased with the increase in the NiS nanosheet content. Moreover, the sample 1.5 wt.% NiS showed the highest production rate of 8654 mL/g·min. The nanocomposite 1.5 wt.% NiS had the highest generation rate among others due to the promising surface design [54]. In this context, NaBH_4_ decomposes in methanol into Na^+^ and ${\mathrm{BH}}_{4}^{-}$ ions. The large surface area of the nanocomposite sample 1.5 wt.% NiS helps the adsorption of more ${\mathrm{BH}}_{4}^{-}$ ions. As a result, the production of hydrogen from methanolysis of NaBH_4_ will be accelerated. Additionally, this rate of hydrogen evolution (8654 mL/g·min) exceeds the rates for R–TiO_2_-NH_2_ (3525 mL/g·min) [50], SiO_2_@PAA (5120 mL/g·min) [55], ZIF-67@GO (3200 mL/g·min) [56], and Ru/NiO-Ni foam (6000 mL/g·min) [57].

## 4. Conclusions

The in situ polycondensation method was implemented for the preparation of S@g-C_3_N_4_ and NiS-g-C_3_N_4_ and was employed for catalytic hydrogen production from the methanolysis of sodium borohydride. The incorporation of NiS during the growth process played a major role in the enhancement of the surface area and porosity of the S@g-C_3_N_4_ composites. The 1.5 wt.% NiS sample had the highest surface area of 90 m^2^/g compared with the 0.5 wt.% NiS and 1.0 wt.% NiS samples. The pore volume of S@g-C_3_N_4_ was 0.18 cm^3^, which was reduced to 0.11 cm^3^ in 1.5 wt.% NiS owing to the incorporation of NiS particles into the nanosheet. We found that during the in situ polycondensation preparation of S@g-C_3_N_4_ and NiS-g-C_3_N_4_ nanocomposites increased the porosity of the composites. The average value of the optical energy gap for S@g-C_3_N_4_ was 2.60 eV and decreased to 2.30 eV because of the 1.5 wt.% NiS incorporation. The NiS-g-C_3_N_4_ catalysts showed an emission band in the 410–540 nm range and the intensity of this peak decreased as the NiS concentration increased from 0.5 to 1.5 wt.%. The hydrogen generation rates increased with the increase in the NiS nanosheet content. The sample 1.5 wt.% NiS showed the highest production rate of 8654 mL/g·min compared with others due to the promising surface design. The large surface area of the nanocomposite sample 1.5 wt.% NiS helps for adsorption of more ${\mathrm{BH}}_{4}^{-}$ ions. As a result, the production of hydrogen from methanolysis of NaBH_4_ will be accelerated. All of these results enhance the possibility of using 1.5 wt.% NiS as a promising catalyst for the production of hydrogen from NaBH_4_ methanolysis.

## Figures and Tables

**Table 1:** Weight % of elements present at the surface of the NiS-g-C_3_N_4_ samples analyzed from EDX analysis.

Sample	C (wt.%)	N (wt.%)	S (wt.%)	Ni (wt.%)
0.5 wt.% NiS	37.75	61.14	0.17	0.94
1.0 wt.% NiS	46.11	52.13	0.30	1.47
1.5 wt.% NiS	35.64	57.27	1.08	6.01

## Data Availability

The corresponding author will make the data available on request.

## Supplementary Materials

The following supporting information can be downloaded at: https://www.mdpi.com/article/10.3390/nano13050938/s1, Figure S1: EDX data for NiS-g-C3N4 nanocomposites.
