# Supplementary material for: In Situ Polycondensation Synthesis of NiS-g-C3N4 Nanocomposites for Catalytic Hydrogen Generation from NaBH4
Source: Nanomaterials (Basel). 2023 Mar 5;13(5):938. doi: 10.3390/nano13050938 (PMC10005517; doi:10.3390/nano13050938)
Supplement: Supplementary file 1 [file nanomaterials-13-00938-s001.zip › nanomaterials-2242214-supplementary.pdf]

Supplementary Materials

In Situ Polycondensation Synthesis of NiS-g-C_3N_4 Nanocomposites for Catalytic Hydrogen Generation from $NaBH_4$

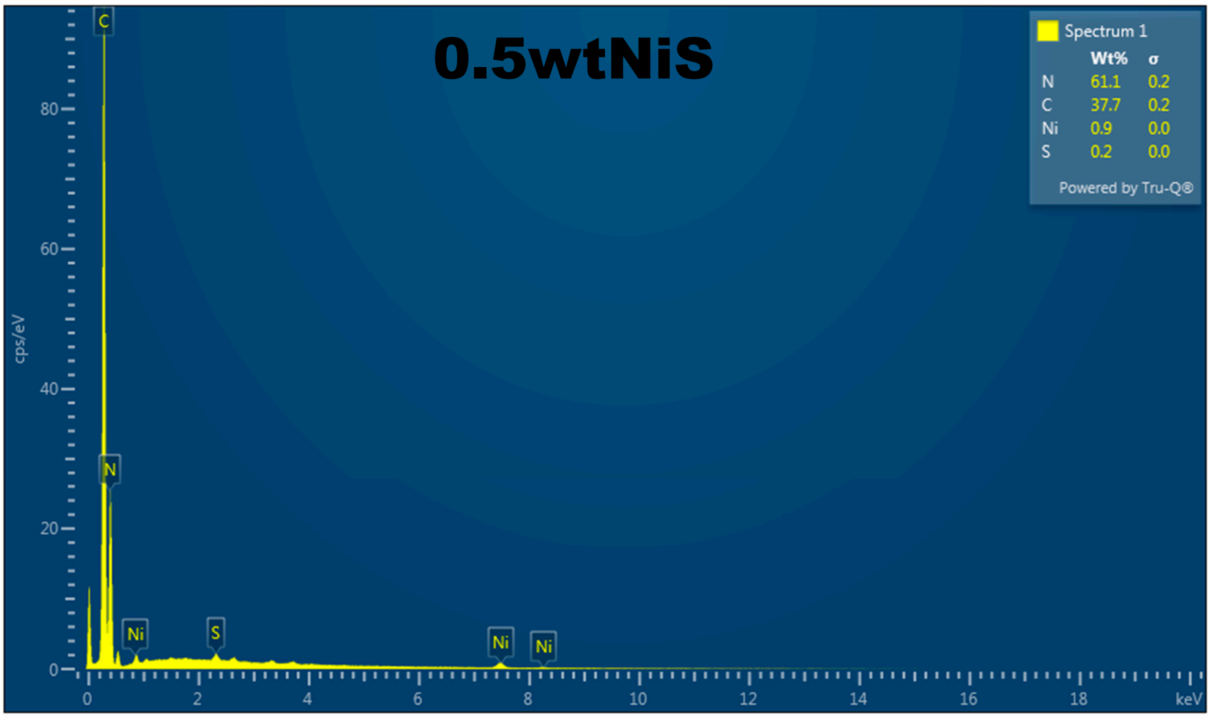

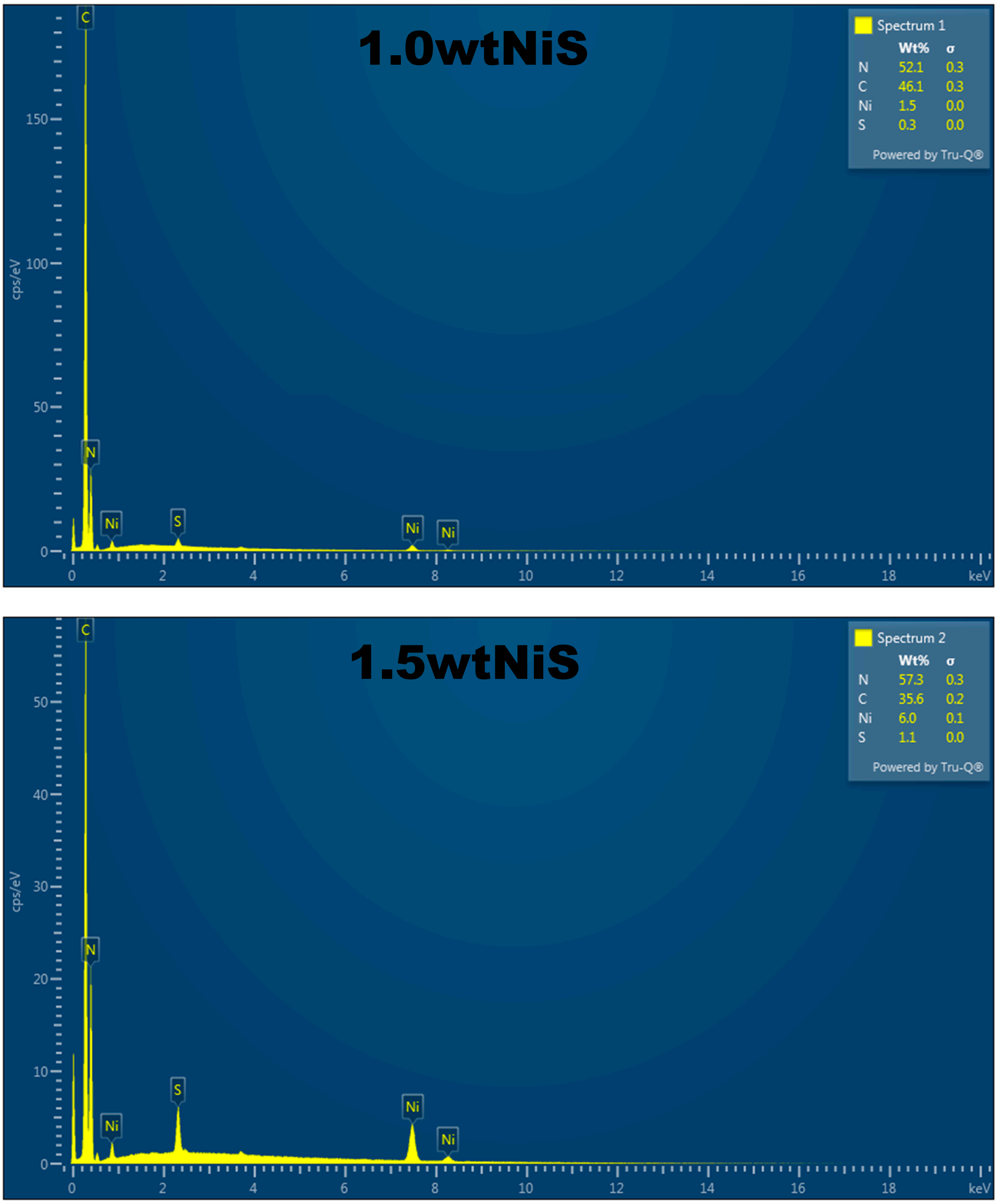

Figure S1. EDX data for NiS-g-C_3N_4 nanocomposites
